# Environmental Management from the Point of View of the Energy Intensity of Road Freight Transport and Shocks

**DOI:** 10.3390/ijerph192114417

**Published:** 2022-11-03

**Authors:** Elżbieta Szaruga, Elżbieta Załoga

**Affiliations:** Department of Transport Management, Institute of Management, University of Szczecin, Cukrowa 8 Street, 71-004 Szczecin, Poland

**Keywords:** energy intensity, environmental management, road freight transport, shocks, vector error correction model

## Abstract

The research aimed to identify the directions of rationalization of the energy intensity of road freight transport in the context of the long-term balance of the drifting economy. The study was related to the case of Poland, and its scope spans 8.5 years. The long-term drift of the economy and shocks from the production process of the construction, industry, and processing sectors were taken into account in the research. In addition, the structural shocks were decomposed and validated. Twenty-one statistically significant multidirectional and varying relationships between the energy consumption of road transport and production in the construction, industry, and processing sectors were confirmed, including 7 long-term and 14 immediate relationships. The leakage of shocks in production has been demonstrated in the construction, industry, and processing sectors. The shock from the construction sector lowered the energy consumption of road transport in the long run. The greatest impact of the shock occurred only after 6–12 months and slowly stabilized after approximately 24 months. On the other hand, the shock in production in the industry and processing sectors reduced energy intensity in the short term but increased in the long term. The shocks in the industry and processing sectors transformed relatively quickly.

## 1. Introduction

### 1.1. Presentation of Research Problems

Environmental management is a planning concept that holistically focuses on the environmental impact of human activities [1]. It deals with the issues of cleaner production and its impact on the environment. It focuses mainly on reducing the negative impact of production processes on the natural environment [2]. It is closely related to the mitigation of atmospheric pollutants, which is considered the challenge of today [3]. Environmental management has been recognized as essential to achieving sustainable development [4]. This also applies to the production activities of transport [5]. One of the main challenges at the center of attention is the decarbonization of the economy [6] and transport [7,8,9], especially when the transport sector generates a significant proportion of global energy-related greenhouse gas (GHG) emissions [7,10,11]. Therefore, the considerations referred to the rationalization of energy consumption in transport and the need for energy management [12], especially since energy is an exhaustive and key resource in the development of humanity [13]. Environmental management mainly tries to find compromise options between the expected state of the environment and the economic, social, technological, or physical limitations of processes [1]. At the same time, attention is paid to the decision-making process, especially at the level of implications of policies referring to the idea of ecological economic growth [14,15] or sustainable development [16]. Environmental management aims to support sustainable development through the prism of an interdisciplinary and holistic approach. Its framework should cover different perspectives (local to global; short-term to long-term) and integrate many different points of view. Its purpose is to improve the integration of ecology, social and economic development, policy making, and planning development directions [1].

Transport is an integral part of the global economy, considered a factor that contributes to many processes and phenomena while participating directly or indirectly in them [17]. Road freight transport plays an important role in the service of transport needs. From 2010 to 2020, road transport in the European Union (EU-27) accounted for 74.6–77.4% of the modal split of inland freight transport, while in Poland this figure was 70.4–77.4% [18]. The energy consumption of road transport in the entire EU-27 economy in 2011–2020 decreased from 27.90% to 26.89% [19,20], i.e., by approximately 1 percentage point. In Poland, the energy consumption of road transport accounted for 26.37% of the energy consumption in the entire economy in 2011, and in 2020, this share increased to 30% [19,20], that is, approximately 4 percentage points. In the EU-27, it can be seen that despite the still growing dominance of road transport in the service of the transport needs of the economy, the global consumption of energy by this mode of transport in total consumption in the analyzed period is decreasing. On the other hand, in Poland, in the years 2010–2020, there is a growing dominance of road transport in servicing the needs of the economy and, at the same time, a growing share of energy consumption by road transport in the global consumption of the entire economy, with road transport accounting for 30% of total energy consumption [19,20]. A similar conclusion could be drawn for previous years. This state and its tendencies justify the need to rationalize the energy consumption of road freight transport as a decision-support tool in environmental management.

This study attempts to identify the directions for the rationalization of the energy intensity of road freight transport, taking into account production shocks in the construction, industry, and processing sectors. The proposed approach to the research problem aligns with the concept of environmental management and sustainable development by integrating the social, ecological, and economic dimensions. The authors filled the cognitive and research gaps in the field of rationalization of the energy intensity of road freight transport from the point of view of dynamic causal relationships that are subject to shocks. The novelty is to take into account the long-term drift of the economy and shocks from the production processes of the complementary sectors: construction, industry, and processing. In their research, the authors emphasized the need to decompose structural shocks, which are reflected in the energy intensity of road freight transport. These studies develop knowledge in the fields of transport economics, transport management, and environmental management. Research has a special cognitive value and contributes to the enrichment of the theory of transport economics and the management of energy intensity in transport. The research may be used by an economic practice or economic policy creator at the stage of policy programming.

### 1.2. Organization of the Paper

The article focuses on examining the impact of shocks in production processes in the construction, industry, and processing sectors on the energy intensity of road freight transport and the assessment of causality in the context of environmental management.

The article presents the research problem in the form of research questions: what should be the directions of rationalization of energy intensity of road freight transport? How do shocks in production processes in the construction, industry, and processing sectors affect the energy intensity of road freight transport? How long does it take to adjust to equilibrium given economic drift and shocks? The research hypothesis is as follows: rationalization of the energy intensity of road freight transport is important and should take into account multiplying shocks in production processes in the construction, industry, and processing sectors, as they affect the adjustment mechanism to the equilibrium level. The article aims to identify the directions for the rationalization of the energy intensity of road freight transport in the context of the long-term balance of the drifting economy. The study was related to the case of Poland. The scope of the study spans 8.5 years. The choice of the time window was deliberate due to the shift in the political and business cycle. Statistical techniques and econometric methods were used, incl. the vector error correction model (VECM) [21,22,23], structural error correction model (SVECM) [24,25], impulse response function (IRF) [26,27,28], Granger causality test [29,30], the Johansen trace test for cointegration [31,32,33], tests to examine the normal distribution (Doornik–Hansen test [34,35], Lütkepohl test [36], and Jarque–Bera test [37,38,39]) and the augmented Dickey–Fuller test [40,41] to investigate stationarity. The choice of these methods meets the need to obtain answers to the above research questions and is necessary for the verification process of undesirable properties and the elimination of apparent dependencies in the context of the conducted study.

The article consists of six parts. The first is an introduction. The second part contains a narrative review of the methodological literature on the application of the vector error correction models to investigate the energy intensity of transport. The third part is a description of the research and methodology. The fourth part contains empirical research results and fifth part—their discussion. The article ends with conclusions.

## 2. Literature Review

There is increasing interest in the subject of transport energy consumption among researchers [42,43,44], especially focused on identifying and mitigating the economic and environmental effects of fuel consumption in transport systems [45,46,47,48]. Concerning research on the relationship between the economic dimension and energy consumption, the research mainly refers to the relationship between economic growth and energy consumption by transport (the research essentially refers to the issue of the decoupling paradigm) [49,50,51]. Zu, Wu, & Peng [52] indicate that in the literature there are four types of research hypotheses about the direction of the relationship between the energy consumption of road freight transport and economic growth. In the first two, a one-way relationship is emphasized (economic growth is the cause of road freight transport or the road freight transport is the cause of economic growth), in the third, feedback is noted, and in the fourth, there is no relationship (neutrality). In verifying the direction of the relationship, they point to the Granger causality test [52]. Nasreen, Mbarek & Rehman [53] identified an additional dimension of dependence, extending it to include environmental quality issues. In a literature review, they identified multidirectional relationships between energy consumption by transport, economic growth, and environmental quality. They focused on pairs of these dependencies. Similarly, they drew attention to the research techniques used by other researchers to analyze these relationships, i.e., the Granger causality test, vector error correction model, and autoregressive-distributed lag [53]. Mohmand et al. [54] note four directions of causality research in the literature. According to them, the first focuses on the relationship between economic growth and investment in transport infrastructure, the second on economic growth and fuel (energy) consumption, the third concerns the relationship between economic growth and environmental pollution, and the fourth is a combination of the three previous approaches. They pay attention to methods for assessing the direction of causality, mentioning such methods as the vector error correction model, environmental Kuznets curve, Johansen cointegration, and autoregressive-distributed lag [54]. The main denominator of all studies is a similar family of econometric methods for investigating causality.

Table 1 presents a methodological review of the literature, which identified the factors, methods, and scope of the study in various approaches to transport (as a whole sector, its modes, or types in horizontal classification). The focus was on works that are similar in terms of research techniques and the scope of the research. The goal was to identify research procedures, including materials and techniques.

**Table 1:** Literature review from a similar methodical point of view.

Transport	Scope	Variables	Methods	Source
freight transport	Tunisia; 1982–2016 (annual)	FT—freight transport in tonne-kilometers; GDP—gross domestic product per capita in constant 2005 USD; EC—energy consumption is expressed in kilotonnes oil equivalent (ktoe); CO_2_—CO_2_ emission is expressed in metric tonnes	VECM to investigate relationships; Augmented Dickey–Fuller test (ADF) and Phillips–Perron (PP) tests for stationarity verification; Johansen cointegration test; Granger causality test; variance decomposition	[55]
total transport sector	30 regions of China; 2004–2016 (annual)	TGDP—transport economic growth in 10^8^ yuan; TEN—transport energy consumption in 10^4^ tonnes standard coal equivalent; TCE—transport CO_2_ emissions in 10^4^ tonnes	VECM to investigate relationships; ADF–Fisher, Levin–Lin–Chu (LLC), and PP tests for stationarity verification; Pedroni panel cointegration tests; FMOLS (fully modified ordinary least squares) to investigate the bidirectional long-run elasticity between variables; Granger causality test	[56]
total transport sector	Thailand; 1990–2017 (annual); forecast for the next 30 years (2018–2047)	CO_2_—carbon dioxide (no information about raw data; used logarithm transformation); GDP—gross domestic product per capita (no information on raw data; used logarithm transformation); L—labor growth (no information about raw data; used logarithm transformation); UR—urbanization rate (no information on raw data; used logarithm transformation); IS—industrial structure (no information about raw data; used logarithm transformation); EC—energy consumption (no information on raw data; used logarithm transformation); FDI—foreign direct investment (no information about raw data; used logarithm transformation); OP—oil price (no information on raw data; used logarithm transformation); X-E—net exports (no information about raw data; used logarithm transformation)	SEM-VECM (structure estimation modeling with an optimization of the vector error correction mechanism model) to investigate relationships; ADF test for stationarity verification; Johansen–Juselius cointegration test	[57]
total transport sector	Pakistan; 1990–2015 (annual)	CO_2_—CO_2_ emissions from transport from fuel combustion (including road, rail, pipeline transport, domestic navigation, and domestic aviation); TRE—transport energy consumption from fossil fuels and gas (in tonnes of oil equivalent); GDP—gross domestic product per capita (constant 2010 USD); FDI—foreign direct investment (% of GDP); URB—urbanization (% of the total population)	VECM to investigate relationships; ADF, PP, and DF-GLS (Dickey–Fuller generalized least square) tests for stationarity verification; ADRL (autoregressive distributed lag model) for cointegration testing; FMOLS, DOLS (dynamic ordinary least squares), and CCR (canonical cointegrating regression) to investigate long-run elasticity between variables; Granger causality test; impulse response function; variance decomposition	[58]
total transport sector	Tunisia; 1980–2007 (annual)	PCGDP—gross domestic product per capita (in constant 2000 USD); PCTFC—transport fuel consumption per capita (in ktoe); PCTCE—transport CO_2_ emissions per capita (in metric tonnes per capita)	VECM to investigate relationships; ADF and PP tests for stationarity verification; Johansen cointegration test; Granger causality test	[59]
total transport sector	71 countries (26 OECD countries * and 45 non-OECD countries **); 1978–2005 (annual)	ENTRA—total energy final consumption for the transport sector (raw data in kg of oil equivalent per capita); used logarithmic transformation; GDP—gross domestic per capita (raw data in constant 2000 USD/capita); used logarithmic transformation; GASPR—total gasoline price (constant 2000 USD/ton of oil equivalent)	VECM to investigate relationships; Levin–Lin–Chu (LLC), Breitung (BRST), Im–Pesaran–Shin (IPS) for stationarity verification; panel v-statistic, panel rho-statistic, panel PP-statistic, ADF-statistic; group rho-Statistic, group PP-statistic, group ADF-statistics for cointegration tests for bi- and multivariate models; Granger causality test	[60]
total transport sector (narrowed to passenger transport)	Tunisia; 1995–2013 (annual)	CO_2_—carbon dioxide emissions from transport (% share of total fuel combustion); Y—real GDP in constant 2005 USD; EUSE—energy use (primary energy use) in kg of oil equivalent; TRS—international tourism (number of tourist arrivals)	VECM to investigate relationships; Levin–Lin–Chu, Breitung, Im–Pesaran–Shin, ADF-Fisher, PP-Fisher tests for stationarity verification; Pedroni cointegration tests; FMOLS and DOLS to investigate long-term elasticity between variables; Granger causality test	[61]
road transport	Egypt; 1980–2011 (annual)	roadec—road energy consumption per capita (kg of oil equivalent); y—real GDP per capita (constant 2005 USD); pop—population growth (annual percentage of population growth); ur—urban population (percentage of the total population)	VECM to investigate relationships; ADF test for stationarity verification; Johansen cointegration test; Granger causality test; impulse response function; variance decomposition	[62]
road transport	US 1946–2006 (annual)	LGDP—real GDP per capita; used logarithmic transformation; LPRICE—real retail gasoline price; used logarithmic transformation; LMFU—motor fuel use per capita; used logarithmic transformation; LVMT—vehicle-miles per capita; used logarithmic transformation; LREG—number of registered vehicles per capita; used logarithmic transformation	VECM to investigate relationships; DF-GLS test for stationarity verification; Johansen cointegration test; Granger causality test	[63]
road freight transport	Poland 2004Q1–2018Q4 (quarterly)	l_EN—energy intensity (raw data expressed as the relation of energy consumption demand in kg per trans- port unit in tkm; kg/tkm); used logarithmic transformation; l_GDP—gross domestic product (raw data expressed in constant prices as an index, 2015 = 100); used logarithmic transformation; l_PPI—index of production prices for energy (raw data expressed as an index, 2015 = 100); used logarithmic transformation	VECM to investigate relationships; ADF test for stationarity verification; Johansen cointegration test; Granger causality test; impulse response function	[64]
road passenger transport	New Zealand; 1990–2016 (annual)	TE—transport emissions (includes GHG emissions from passenger land transport, in MtCO_2_e); PV—numbers of light passenger vehicles (millions); P—fuel price (real annual average petrol prices including the effect of the emissions trading scheme (ETS) on prices; New Zealand cents/liter in constant price to 2017); FE—vehicle fuel economy (total distance traveled by a light petrol vehicle, divided by amount; in km/L); GDP—gross domestic product per capita (constant 2010 USD) U—level of urbanization (percentage of the population living in urban areas; in %)	VECM to investigate relationships; ADF and PP tests for stationarity verification; Johansen cointegration test; Granger causality test	[65]
urban passenger transport	Pakistan; 1972Q1–2011Q4 (quarterly)	EC—energy consumption per capita (kg of oil equivalent); U—urban population per capita; A—affluence (wealth or prosperity; proxied by real GDP/capita); TEC—technology per capita (proxied by interaction term of industry and services sectors value-added); TP—use of transport per capita per km (proxied by the number of cars and buses)	VECM to investigate relationships; Narayan and Popp (NP), ADF, and PP tests for stationarity verification; ADRL for cointegration testing; Granger causality test	[66]
urban transport	Jakarta (Indonesia); 2001–2014 (annual)	LNTOT—total transport energy use (raw data probably in liters); used logarithmic transformation LNPOP—total urban population (raw data probably in persons; proxied to urbanization); used logarithm transformation	VECM to investigate relationships; Levin–Lin–Chu, Im–Pesaran–Shin, ADF-Fisher, PP-Fisher test for stationarity verification; Granger causality test	[67]

Notes: * 26 OECD (Organisation for Economic Cooperation and Development) countries: Australia, Austria, Belgium, Canada, Denmark, Finland, France, Germany, Greece, Hungary, Iceland, Ireland, Italy, Japan, Korea Rep., Mexico, Netherlands, New Zealand, Norway, Portugal, Spain, Sweden, Switzerland, Turkey, United Kingdom, United States; ** 45 non-OECD countries: Algeria, Argentina, Bolivia, Brazil, Cameroon, Chile, China, Colombia, Congo Dem. Rep., Costa Rica, Cote d’Ivoire, Ecuador, Egypt, Gabon, Ghana, Guatemala, Honduras, India, Indonesia, Iran, Israel, Jamaica, Jordan, Kenya, Malaysia, Morocco, Nepal, Nigeria, Pakistan, Paraguay, Peru, Philippines, Romania, Saudi Arabia, Senegal, Singapore, Sout Africa, Sri Lanka, Sudan, Syrian Arab Rep., Thailand, Tunisia, Uruguay, Venezuela, Zimbabwe. Source: own elaboration based on sources from the last column of the table.

The methodological overview presented in Table 1 shows the analytical macro context of energy consumption in transport. However, an approach to the macroeconomic and mesoeconomic (intersectoral) interface can also be found. In addition to the above-mentioned studies, the literature mentions the intersectoral relationship between energy consumption by transport and production in other sectors [68]. Wang et al. [69] examined the cross-sectoral relationship in terms of carbon dioxide emissions (noting that it comes from energy consumption: electricity and fuel) between transport and other sectors of the economy, e.g., energy industry; chemicals, non-fossil fuel mining, and products; agriculture; food, clothing, timber, and other manufacturing industry; service; construction; electromechanical manufacturing and high technology industry. Other scientists have also researched the links between energy consumption in transport and other sectors. Bejany, Zhang, and Xia proposed a model to minimize the energy consumption of trucks in open-cast mining, but this is a static approach, referring to technical parameters on a microscale [70]. Wang et al. addressed a similar topic, pointing to the need to identify truck consumption patterns in the open-cast industry. Using the experimental method, they analyzed energy consumption in 5179 transport cycles [71]. Whyte, Daly & Gallachóir examined Ireland’s energy demand for road freight transport (heavy goods vehicles) by commodity groups and then linked them to economic factors such as GDP, and gross value added (separately in construction and industry). They evaluated the effects of the building bubble, which incidentally contributed to the shocks to the economy [72]. It is also emphasized that the demand shock has a significant impact on energy intensity in road transport, much greater than the supply factors of transport services [72], as also the COVID-19 pandemic showed. Undoubtedly, shocks from production in sectors complementary to transport, that is, construction, industry, and processing, also demand shocks for transport services.

## 3. Data and Methods

### 3.1. The Scope of the Study

The identification of the links between rationalization of the energy intensity of road transport and production in the construction, industry, and processing sectors was carried out based on monthly time series for Poland. Data come from the OECD database [73]. The study covers the period from January 2008 to June 2016. The selection of the time range is deliberate due to economic selectors and requires a broader justification in the context of the economic situation in Poland.

The research period can be divided into so-called time windows based on certain criteria. Generally, it is possible to isolate three time perspectives for analysis for Poland after joining the European Union: the first—2004–2007, the second—2008 until June 2016, and the third—from July 2016 to the present day. The last window of time could be split into two separate windows due to the emergence of the COVID-19 pandemic crisis.

The period 2004–2007 is a time of permanent adaptation of legislation to the law of the European Union (EU), launching financial instruments from EU funds, which were to support the goals of sustainable development and stimulate the Polish economy. One of the goals of these activities was to stimulate the economy, including transport activity, and to use the potential of other sectors of the economy (e.g., construction, industry, processing). On the other hand, the need to minimize the impact of economic growth on the environment was emphasized (in line with the shift paradigm, i.e., in transport [17,74,75]). One of the most important challenges was rationalizing the energy intensity of the economy, especially transport. The impact of EU policy and funds was particularly visible in transport activity, which is also reflected in changes in the energy intensity of the entire transport sector and its modes, especially road transport. Furthermore, the time range 2004–2007 is characterized by an atypical nature; that is, it is an outlier compared to the entire period 2004–2022. For the period 2004–2007, atypical observations are noticeable in connection with structural changes, economic transformation, and integration, as well as other temporary changes. The years 2004–2007 can be described as changes resulting from Poland’s integration with the EU. This period was omitted from the analysis due to unusual patterns of data shaping.

In 2007, there was a global financial crisis that became the global economic crisis of 2008. However, it should be noted that Poland was overshadowed by this crisis. Therefore, the beginning of 2008 was considered appropriate for an analysis of the example of Poland. First, it concerns the prosperity of transport activity in Poland [76]. This prosperity is understood through the prism of significant increases in the results of transport activities in Poland compared to the previous year, an increase in the volume of road freight transport by 10%, and transport performance by approximately 9% [76]. At the same time, the increase in transport activity placed Poland in the first position in international transport of the European Union and the sixth position in general road freight transport [76]. Second, it refers to the relatively smooth transition of Poland through the global economic crisis until the new economic situation associated with positive shocks that stimulated the development of the Polish economy. The stimulation of economic development took place, on the one hand, thanks to EU programs, and on the other, in a later perspective (the second half of 2016)—by initiating internal demand with social programs, that is, “Rodzina 500+” (“Family 500+”) [77]. The following years are the time when negative shocks appear [78]. Therefore, the period after June 2016 to 2022 requires the analyzed dependencies to be separated from the period from January 2008 to June 2016, more so because they relate to completely different business and political cycles.

The years 2019–2022 are a period of the COVID-19 pandemic crisis (mainly associated with lockdown). In addition, the beginning of 2022 is associated with the Russia–Ukraine war crisis, which also affects Poland. Both crises multiply and overlap. One should also remember the fuel price shock, first in 2021, then in 2022, which would be significantly reflected in the analysis of energy consumption in transport and production in the construction, industry, and processing sectors. The unfavorable economic situation in Poland in the current period, especially related to the multiplication of negative demand-supply, price, and social shocks along with rising inflation, could significantly distort the image of the relationships analyzed. These are mainly external shocks, but also internal shocks. Internal shocks are conditioned by the economic situation, i.e., the introduction of a new order (the “Polish Deal” [79,80]) and significant increases in interest rates (as a tool for the impact of monetary policy), which redefined the rationality of economic entities. The solidification of these changes and the closure of certain time frames will allow the comparison of two time perspectives, assigned to completely different phases of the business cycle (strongly related to the economic situation, and the formation of market relations) and, in a way, the political cycle (strongly linked to economic and sectoral policies, directions and instruments of influencing market relations). Therefore, the scope of the study was contractually closed in June 2016.

Due to the need to identify the relationship between road transport and increased production in the construction, industry, and processing sectors in the era of prosperity of transport, empirical values fill the gap in shaping the energy consumption of road transport in relation to increased production in other sectors of the economy. At the same time, they are the basis for comparisons in the period when economic activity was subject to negative shocks that favor the risk of stagnation of the economy.

### 3.2. Data and Techniques Description

The analyzed data covered the period from January 2008 to June 2016 (time series) and came from the OECD database [73]. The variables used included in the study were represented by:

- EN—energy intensity of road freight transport, where the energy intensity of road freight transport was expressed as total fuel supplies to the transport sector in relation to transport performance (expresses the volume of fuel supplies in tons for the performance of a road freight transport unit in tkm); raw data in t/tkm,
- Qc—production in construction (index 2010 = 100),
- Qp—production in processing (manufacturing) (index 2010 = 100),
- Qi—production in industry (index 2010 = 100),
- l_EN—logarithmic energy intensity of road freight transport,
- l_Qc—logarithmic production in construction,
- l_Qp—logarithmic production in processing,
- l_Qi—logarithmic production in the industry.

Data on production in other sectors of the economy were not taken into account due to the specificity (e.g., for another type of transport—pipeline) or did not have appropriate properties for the methodology used.

All time series were analyzed for stationarity using the ADF test [81,82], taking into account the optimal number of lags. The optimal number of lags was determined based on the information criteria of the final prediction error (FPE) [83,84], Akaike (AIC) [85,86], Schwarz (SC) [87], and Hannan–Quinn (HQ) [88,89,90]. If the stationarity of the time series was not confirmed, the study was supplemented with a cointegration test, the so-called Johansen trace test [33,82,91]. On its basis, it was determined whether the series were stationary or not and what form (unlimited/limited) of the model would be appropriate: vector autoregressive model (VAR) or vector error correction model (VECM). If it was necessary to construct an unlimited VAR model, the analysis was supplemented with an extended Johansen cointegration study. Based on the extended analysis, it was possible to obtain standardized matrices of adjustments and cointegrations, by which a record of equilibrium equations was obtained. When choosing VECM, restrictions were imposed on the specification using the SER (sequential elimination of regressors) system, taking into account HQ. The estimation of the model made it possible to distinguish the long-term equilibrium equation and assess the mechanism of adjustment to the equilibrium level, as well as examine the Granger causality [92,93,94]. Each time, the estimated model was verified in terms of the presence of causal relationships using the Granger causality test. Based on the results obtained, a dynamic relational model of the energy intensity of road freight transport and other variables studied was presented. The models were then verified for the desired properties: normal distribution of residuals (Doornik–Hansen test; Lütkepohl test; and Jarque–Bera test); no autocorrelation between residuals (LM-TYPE test), and no ARCH effect (ARCH-LM test and multivariate ARCH-LM test).

Subsequently, the model was corrected to the structural form of the SVECM B-Model. Based on the estimated structural forms, it was possible to study the impulse response function (IRF). The identification of shocks was necessary to assess their impact on the energy intensity of road freight transport and, at the same time, to assess the impact of shocks resulting from the energy intensity of this type of transport on other variables.

Selected descriptive statistics are presented in Table 2.

As shown in Table 2, the largest level of variation, i.e., the quotient of the standard deviation and the mean, is characteristic for production in construction, but for industry, processing sectors, and the energy intensity of road freight transport, the values are similar. As the variables are logarithmic, smaller differentiation of the examined variables can be noticed. In all analyzed cases, there is a higher value for the median than the mean, which means that more than half of the studied observations achieved a value greater than the mean, but the differences are relatively small, which does not indicate that the variables should be assessed through the prism of their asymmetry (left-hand skew). Due to the specific functional form of the model (multiplicative), in the subsequent part of the study, logarithmic forms of variables were included in the analysis. Transformation of the variables into their logarithmic forms is necessary from the point of view of the desired features of the variables and the model.

All variables were examined for the presence of unit roots. The study was carried out using the augmented Dickey–Fuller test, taking into account the occurrence of drift, trend, and seasonal variables (Table 3).

As shown in the data in Table 3, the logarithmic series are not stationary with an optimal number of lags. Because the stationarity of the series was not confirmed, a cointegration study was performed using the Johansen trace test (Table 4).

From Table 4 it can be seen that the cointegrating relations do not exist at the order r0 = 0 (they are statistically insignificant *p* < 0.05, and LR = 74.62 is above the 99% threshold). On this basis, consecutive orders of cointegration should be considered. With the first order of cointegration (r0 = 1), it can be assumed that there are significant cointegrating relations (the hypothesis that there are no such relations for this row cannot be rejected; *p*-value > 0.05, and LR = 35.20 is below the 90% threshold). Cointegrating relations also exist for the 2nd and 3rd order of cointegration, but the lowest one is assumed.

### 3.3. Specification of Models in a Non-Structured and Structured Form

The data presented in Table 4 suggest that there is one long-term cointegrating relationship between the variables included in the study (taking into account the third order of lags). Therefore, the specification, estimation, and verification of VECM instead of VAR are justified. VAR can be used when variables are stationary or when in the cointegration study the Johansen footprint test indicates a zero or a full row of the matrix Π [95].

The model was specified based on the tests to verify the series stationarity and cointegration. Johansen’s procedure was used with restrictions imposed by the SER system (based on the HQ results):

(1) $$\left[\begin{array}{c} \Delta l\_{\mathrm{EN}}_{t}\\ \Delta l\_{\mathrm{Qc}}_{t}\\ \Delta l\_{\mathrm{Qi}}_{t}\\ \Delta l\_{\mathrm{Qp}}_{t} \end{array}\right]=\left[\begin{array}{c} {}*\\ {}*\\ {}*\\ {}* \end{array}\right]\left[\begin{array}{c} \mathrm{EC}1_{t-1} \end{array}\right]+\left[\begin{array}{cccc} {}* & 0 & 0 & *\\ 0 & * & * & *\\ {}* & 0 & * & *\\ 0 & 0 & * & * \end{array}\right]\left[\begin{array}{c} \Delta l\_{\mathrm{EN}}_{t-1}\\ \Delta l\_{\mathrm{Qc}}_{t-1}\\ \Delta l\_{\mathrm{Qi}}_{t-1}\\ \Delta l\_{\mathrm{Qp}}_{t-1} \end{array}\right]+\left[\begin{array}{cccc} 0 & 0 & 0 & *\\ 0 & * & * & *\\ 0 & * & * & 0\\ 0 & * & * & 0 \end{array}\right]\left[\begin{array}{c} \Delta l\_{\mathrm{EN}}_{t-2}\\ \Delta l\_{\mathrm{Qc}}_{t-2}\\ \Delta l\_{\mathrm{Qi}}_{t-2}\\ \Delta l\_{\mathrm{Qp}}_{t-2} \end{array}\right]$$

where ∗ means inclusion in the model, 0 means exclusion from the model, Δ means the first differences, the lower index t means the current month, t − 1 the previous month, and t − 2 a shift by 2 months back.

The optimal number of lags for VECM was determined using the following information criteria: AIC, HQ, SC, and FPE. It is 2.

Based on the analysis of the directions of connections between energy intensity and production in the construction, industry, and processing sectors, the diagnosis of structural shocks in the structural-dynamic relational model was carried out. For this purpose, the VECM model (vector error correction model) was corrected to the SVECM (structural vector error correction model) form. Correction to the SVECM model was carried out based on the B-model estimation with long-term restrictions. The specification of matrix B and the long-term interaction matrix are presented in Table 5.

The specification presented shows that no real restrictions were imposed on long-term relations, but six restrictions were imposed on current relations (zero constraints). The adopted model specification was used for estimation with the ML method, using the scoring algorithm [96].

The estimation results are presented in Section 4.

## 4. Results

### 4.1. Dynamic Relational Model

When specifying the variables, the model included a limited drift, a limited trend, and limited seasonal variables, treating them as conditions that limit the cointegration relationship.

The results of the matrix estimation are presented below.


(2) $$\begin{array}{l} \left[\begin{array}{c} \Delta l\_{\mathrm{EN}}_{t}\\ \Delta l\_{\mathrm{Qc}}_{t}\\ \Delta l\_{\mathrm{Qi}}_{t}\\ \Delta l\_{\mathrm{Qp}}_{t} \end{array}\right]=\left[\begin{array}{c} 0.00476\\ 0.03882\\ 0.00094\\ -0.00027 \end{array}\right][\left[\begin{array}{cccc} 1.000 & -3.768 & -29.411 & 32.927 \end{array}\right]\left[\begin{array}{c} l\_{\mathrm{EN}}_{t-1}\\ l\_{\mathrm{Qc}}_{t-1}\\ l\_{\mathrm{Qi}}_{t-1}\\ l\_{\mathrm{Qp}}_{t-1} \end{array}\right]\\ \;+\begin{array}{ccccccc} {}[-11.262 & 24.225 & 21.172 & 22.033 & 22.785 & 23.901 & 21.817\\ 23.187 & 22.496 & 24.408 & 21.644 & 33.025 & -0.028 & ] \end{array}\;\cdot [\begin{array}{c} \mathrm{CONST}\\ S1_{t-1}\\ S2_{t-1}\\ S3_{t-1}\\ S4_{t-1}\\ S5_{t-1}\\ S6_{t-1}\\ S7_{t-1}\\ S8_{t-1}\\ S9_{t-1}\\ S10_{t-1}\\ S11_{t-1}\\ {\mathrm{TREND}}_{t-1} \end{array}]]\\ \;+[\begin{array}{cccc} -0.344 & 0.129 & -0.531 & 0.354\\ 0.000 & -0.039 & -4.079 & 3.608\\ -0.011 & -0.037 & -0.746 & 0.346\\ -0.004 & -0.054 & -1.558 & 1.088 \end{array}][\begin{array}{c} \Delta l\_{\mathrm{EN}}_{t-1}\\ \Delta l\_{\mathrm{Qc}}_{t-1}\\ \Delta l\_{\mathrm{Qi}}_{t-1}\\ \Delta l\_{\mathrm{Qp}}_{t-1} \end{array}]\\ \;+[\begin{array}{cccc} -0.040 & 0.003 & 2.031 & -1.940\\ -0.056 & -0.163 & -3.651 & 3.334\\ 0.070 & -0.099 & -2.283 & 1.828\\ 0.071 & -0.110 & -2.662 & 2.188 \end{array}][\begin{array}{c} \Delta l\_{\mathrm{EN}}_{t-2}\\ \Delta l\_{\mathrm{Qc}}_{t-2}\\ \Delta l\_{\mathrm{Qi}}_{t-2}\\ \Delta l\_{\mathrm{Qp}}_{t-2} \end{array}]+[\begin{array}{c} u1_{t}\\ u2_{t}\\ u3_{t}\\ u4_{t} \end{array}] \end{array}$$

Based on the components of the normalized cointegration vector of the VECM model, it is possible to calculate the excess energy intensity of road transport based on the equation of the long-term equilibrium between the energy intensity of road transport, production in the construction, industry, and processing sectors:

(3) $$\begin{array}{l} l\_{\mathrm{EN}}_{t}=3.768\;l\_{\mathrm{Qc}}_{t}+29.411\;l\_{\mathrm{Qi}}_{t}-32.927\;l\_{\mathrm{Qt}}_{t}+11.262\;\mathrm{CONST}\\ \;-24.225\;S1_{t}-21.172\;S2_{t}-22.033\;S3{\;}_{t}-22.785\;S4_{t}\\ \;-23.901\;S5_{t}-21.817\;S6_{t}-23.187\;S7_{t}-22.496\;S8_{t}\\ \;-22.408\;S9_{t}-21.644\;S10_{t}-33.025\;S11_{t}+0.028{\;\mathrm{TREND}}_{t} \end{array}$$

As Equation (3) shows, the elasticity of the energy intensity of road transport in relation to production in construction is approximately 3.8, while in relation to production in the industry sector, it is approximately 29.4. In addition, in the processing sector, it is around 32.9. However, the elasticities of the energy intensity of road transport in relation to production in the industry and processing sectors are not statistically different from zero, and taking into account these variables was necessary due to delayed endogenous conditions. In other words:

- an increase in construction production of 1% contributes to an increase in the energy intensity of road transport of 3.8% *ceteris paribus*;
- an increase in industrial production of 1% contributes to an increase in the energy intensity of road transport of 29.4% *ceteris paribus*;
- and an increase in processing production of 1% contributes to a decrease in the energy intensity of road transport of 32.9% *ceteris paribus*.

Therefore, there is weak sustainability between the energy intensity of road transport and production in the construction industry and strong sustainability between the energy intensity of road transport and production in the industry and processing sectors.

Based on the vector α, it is possible to assess the speed of adaptation of the energy intensity of road transport to the long-term equilibrium after the disturbance of the equilibrium state (Equations (4)–(7)):

(4) $$\begin{array}{l} \Delta l\_{\mathrm{EN}}_{t}=0.00476\;(3.768{\;l}_{{\mathrm{Qc}}_{\text{t}-1}}+29.411{\;l}_{{\mathrm{Qi}}_{\text{t}-1}}-32.927{\;l}_{{\mathrm{Qp}}_{\text{t}-1}}\\ \;+11.262\text{ CONST }-24.225\text{ S}1_{\text{t}-1}\;-21.172\text{ S}2_{\text{t}-1}\\ \;-22.033\text{ S}3{\;}_{\text{t}-1}-\;22.785\text{ S}4_{\text{t}-1}\;-23.901\text{ S}5_{\text{t}-1}\\ \;-21.817\text{ S}6_{\text{t}-1}-23.187\text{ S}7_{\text{t}-1}-22.496\text{ S}8_{\text{t}-1}\\ \;-22.408\text{ S}9_{\text{t}-1}\;-\;21.644\text{ S}10_{\text{t}-1}\;-\;33.025\text{ S}11_{\text{t}-1}\\ \;+0.028{\text{ TREND}}_{\text{t}-1})\;+\;\ldots \end{array}$$

(5) $$\begin{array}{l} \Delta l\_{\mathrm{Qc}}_{t}=0.03882\;(3.768{\;l}_{{\mathrm{Qc}}_{\text{t}-1}}+29.411{\;l}_{{\mathrm{Qi}}_{\text{t}-1}}-32.927{\;l}_{{\mathrm{Qp}}_{\text{t}-1}}\\ \;+11.262\text{ CONST }-24.225\text{ S}1_{\text{t}-1}\;-21.172\text{ S}2_{\text{t}-1}\\ \;-22.033\text{ S}3{\;}_{\text{t}-1}-\;22.785\text{ S}4_{\text{t}-1}\;-23.901\text{ S}5_{\text{t}-1}\\ \;-21.817\text{ S}6_{\text{t}-1}-23.187\text{ S}7_{\text{t}-1}-22.496\text{ S}8_{\text{t}-1}\\ \;-22.408\text{ S}9_{\text{t}-1}\;-\;21.644\text{ S}10_{\text{t}-1}\;-\;33.025\text{ S}11_{\text{t}-1}\\ \;+0.028{\text{ TREND}}_{\text{t}-1})\;+\;\ldots \end{array}$$

(6) $$\begin{array}{l} \Delta l\_{\mathrm{Qi}}_{t}=0.00094\;(3.768{\;l}_{{\mathrm{Qc}}_{\text{t}-1}}+29.411{\;l}_{{\mathrm{Qi}}_{\text{t}-1}}-32.927{\;l}_{{\mathrm{Qp}}_{\text{t}-1}}\\ \;+11.262\text{ CONST }-24.225\text{ S}1_{\text{t}-1}\;-21.172\text{ S}2_{\text{t}-1}\\ \;-22.033\text{ S}3{\;}_{\text{t}-1}-\;22.785\text{ S}4_{\text{t}-1}\;-23.901\text{ S}5_{\text{t}-1}\\ \;-21.817\text{ S}6_{\text{t}-1}-23.187\text{ S}7_{\text{t}-1}-22.496\text{ S}8_{\text{t}-1}\\ \;-22.408\text{ S}9_{\text{t}-1}\;-\;21.644\text{ S}10_{\text{t}-1}\;-\;33.025\text{ S}11_{\text{t}-1}\\ \;+0.028{\text{ TREND}}_{\text{t}-1})\;+\;\ldots \end{array}$$

(7) $$\begin{array}{l} \Delta l\_{\mathrm{Qp}}_{t}=0.00027\;(3.768{\;l}_{{\mathrm{Qc}}_{\text{t}-1}}+29.411{\;l}_{{\mathrm{Qi}}_{\text{t}-1}}-32.927{\;l}_{{\mathrm{Qp}}_{\text{t}-1}}\\ \;+11.262\text{ CONST }-24.225\text{ S}1_{\text{t}-1}\;-21.172\text{ S}2_{\text{t}-1}\\ \;-22.033\text{ S}3{\;}_{\text{t}-1}-\;22.785\text{ S}4_{\text{t}-1}\;-23.901\text{ S}5_{\text{t}-1}\\ \;-21.817\text{ S}6_{\text{t}-1}-23.187\text{ S}7_{\text{t}-1}-22.496\text{ S}8_{\text{t}-1}\\ \;-22.408\text{ S}9_{\text{t}-1}\;-\;21.644\text{ S}10_{\text{t}-1}\;-\;33.025\text{ S}11_{\text{t}-1}\\ \;+0.028{\text{ TREND}}_{\text{t}-1})\;+\;\ldots \end{array}$$

Based on the estimates of the vector α, it can be seen that the production in construction and the energy intensity of road transport are adjusted to the equilibrium equation of energy intensity in road transport, while the rate of adjustment of production in construction is higher than the rate of adjustment of the energy intensity of road transport alone. The equilibrium adjustment amounts to approximately 3.88% and 0.48%, respectively, within a month. The return to the equilibrium level in the case of production in the construction industry takes approx. 2 years and 1 month, and in the case of energy intensity of road transport—approx. 17 years and 4 months. Adjustment parameters in the equations of production dynamics in the industry and processing sectors do not differ statistically from zero. This means that there is a weak exogeneity of these two variables with the cointegrating relationship, i.e., adjustments to the equilibrium level occur from the side of factors shaping dynamics of production in the industry and processing sectors, and not through production itself in those sectors. This conclusion is important for the entire system.

The estimated model was characterized by the desired properties. The residuals of the model were normally distributed, there was no univariate or multivariate ARCH effect, and the residuals did not show autocorrelation. No additional restrictions were imposed on the vector β as the model lost the desired properties.

Based on the estimated model, a Granger causality analysis was performed to identify causal relationships between the variables (Table 6).

Table 6 attempts to identify 28 cause–effect relationships, including 14 general Granger causalities and 14 immediate Granger causalities. As a result of the research carried out using the Granger causality test, the existence of 21 different cause–effect relationships was verified: 7 general and 14 immediate.

**Figure 1:**
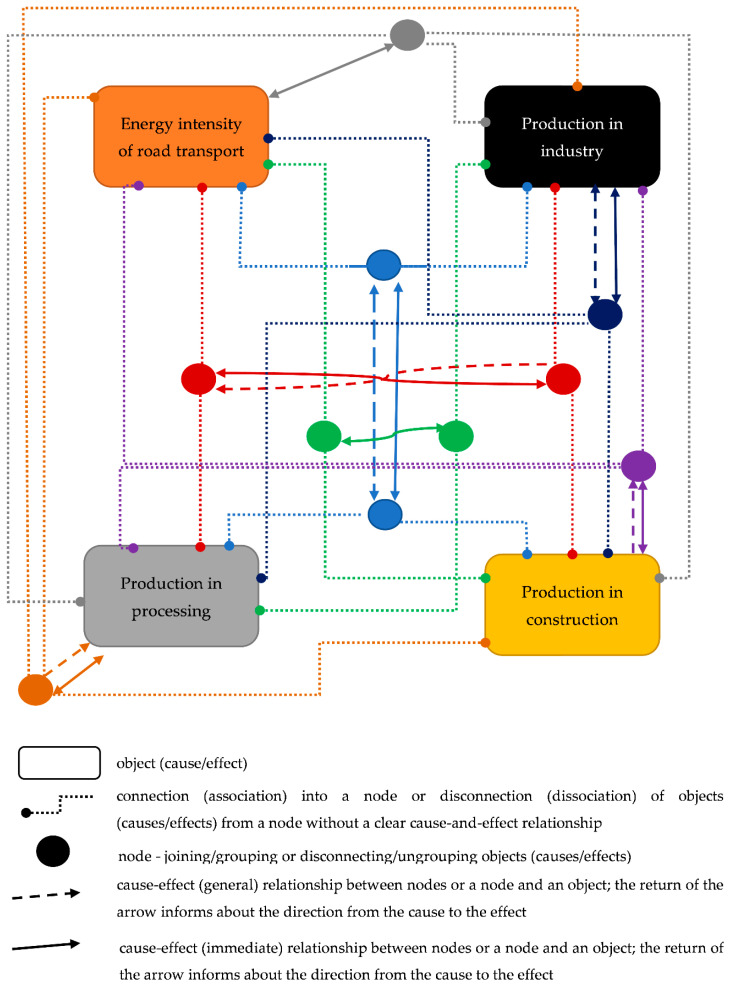
The dynamic relational model of the energy intensity of road transport and production in the construction, industry, and processing sectors. Source: own elaboration based on the results presented in Table 6.

Based on the results obtained in Table 6, a dynamic relational model of energy intensity is illustrated in road transport, and production in the construction, industry, and processing sectors (Figure 1).

As shown in Figure 1, there are complex and multidirectional relationships between the energy intensity of road transport, and production in the construction, industry, and processing sectors. Diverse relations can be observed not only at the level of the influence of one variable on another but also at the level of the association of variables influencing the association of other variables, which allows for the capture of the synergy effects. It is worth noting also the feedback between the nodes, which represents the two-way interaction of associations of variables. Just like between nodes and variables.

Figure 1 illustrates not only the complexity of the relationships analyzed, but also the complexity of rationalizing the energy of road transport without damaging production in the construction, industry, and processing sectors, where road transport plays an important role. The analyzed relationships illustrate the sensitivity of decision-making, burdened with uncertainty on the one hand, and with a wave of secular stagnation on the other.

### 4.2. Structural Dynamic Shocks Model

An extension of the results presented in the previous subsection is the presentation of shocks in the analyzed system of relations. This is possible due to the structural form of the model described (the specification is presented in Section 3.3) The SVECM estimation results are below (Table 7):

The correction of the VECM models to the SVECM form is mainly performed for the approximation of the impulse response function (IRF). Structural forms of models are consistent with the assumptions of economic theories and can be a tool used in economic policy [95].

**Figure 2:**
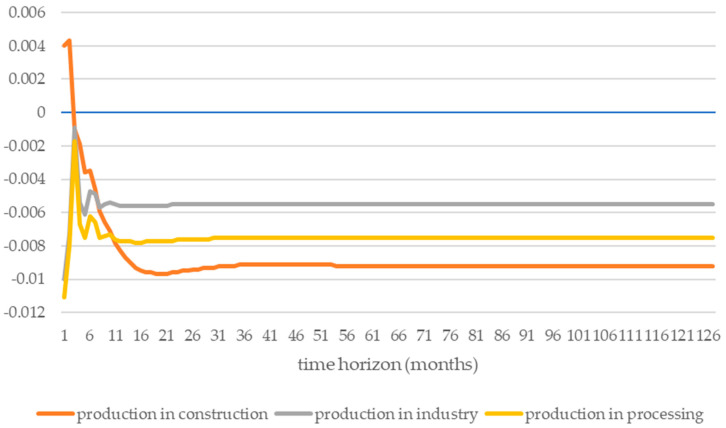
Changes in production in the construction, industry, and processing sectors as a result of shocks caused by the energy intensity of road transport. Source: own elaboration based on data from the OECD database [73].

**Figure 3:**
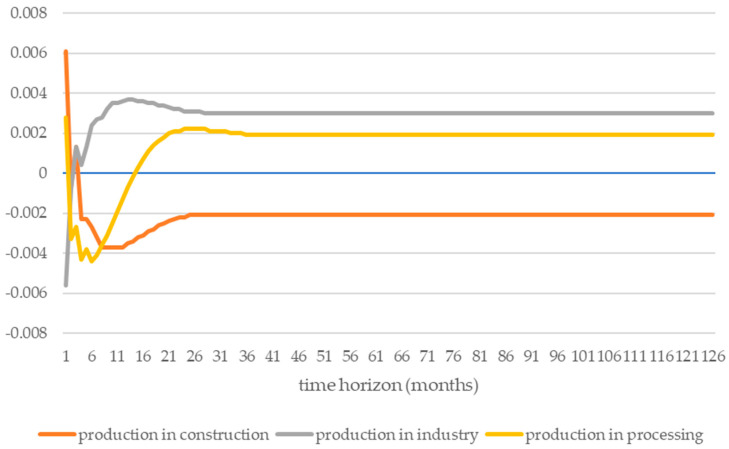
Changes in the energy intensity of road transport are under the influence of shocks from production in the construction, industry, and processing sectors. Source: own elaboration based on data from the OECD database [73].

The impulse response function in two directions was investigated. The first direction of the impulse focuses on the response to production in the construction, industry, and processing sectors under the influence of exogenous shocks from the energy intensity of road transport (Figure 2). The second direction is represented by the reactions of the energy intensity of road transport to endogenous shocks from production in the construction, industry, and processing sectors (Figure 3).

The analysis of Figure 2 shows that only in the construction industry in the first month was there a positive energy intensity shock; in the following months, it transformed into a negative form of shock, achieving a short-term equilibrium. In the case of the industry and processing sectors, the shock from the energy intensity of road transport was an exogenous negative shock that had negative effects on production in these sectors of the economy. It seems that the shock of energy intensity in road transport affects production in the industry with a slight delay (about 1 month). This could indicate a shock leakage effect. Moreover, after some time, these shocks remain at a constant and low level, which proves the stability of the modeled system. On the other hand, collapse in the transport activity sphere (expressed by the energy consumption of road transport) was found to cause long-term declines in the construction, industry, and processing sectors. In a short period (up to the first quarter), this shock was caused by an increase in production in the construction sector. Unfortunately, in a time horizon longer than a quarter, it caused the most significant decline in other sectors.

In the case of the analysis of the impact of shocks from production in the construction, industry, and processing sectors on the energy intensity of road transport (Figure 3), it can be seen that a shock from production in construction affects the energy intensity of road transport, as does the shock of energy intensity of road transport in production in construction, perhaps due to the exogeneity of these two variables to each other. However, the shock of production in construction was positive in the initial period and then turned into a negative shock, reaching a short-term equilibrium. On the other hand, shocks from production in the industry and processing sectors show the opposite situation; they transform from negative shocks to positive shocks, passing through a short-term equilibrium. There is a shift of approximately 6 months between these shocks. These shocks stabilize after approx. 2 years, which proves the stability of the modeled system. In the long run, the shock from the construction sector reduced the energy consumption of road transport. On the other hand, a shock in production in the industry and processing sectors decreased energy intensity in the short term but increased in the long term. The shocks in the industry and processing sectors transformed relatively quickly. The shock from the processing sector was unusual (dichotomous)—in the first year it reduced energy intensity, and after 18 months it turned into a shock causing energy intensity increases. The shock from the industry sector was similar, but the shock transformation occurred much faster after the first quarter.

## 5. Discussion

The rationalization of the energy consumption of road transport was diagnosed as not a straightforward one-dimensional effect. It entails several consequences, which are imposed by multiplying shocks with very long-lasting effects, which affect production in the construction, industry, and processing sectors. The aim of the research has been achieved. The hypothesis has been positively verified and confirmed. When trying to answer the research questions, it should be noted that the rationalization directions of energy consumption should take into account the consequences for the sectors that are stimulated by transport and should not be at the expense of inhibiting transport or production activity in the sectors analyzed. In determining the directions of rationalization, beyond the short perspective, which may be deceptive, one should take into account the long-term horizon with the probability of the occurrence of multiplying and transforming shocks. The shocks in the analyzed sectors do not have an identical pattern of impact on the energy consumption of transport. The shock to production in construction initially causes an increase in the energy consumption of transport in the very short term, but it quickly transforms and in the long term results in a reduction in the energy consumption in transport. The reverse direction presents an industrial shock. In the short term, it can contribute to the reduction in energy consumption, but in the long term, it is disruptive and increases the energy consumption of road freight transport. The shock in the production in the processing sector looks similar. The shocks from the industry and processing sectors shift towards each other for about six months. As a result of the drift of the economy and the impact of short- and long-term relations and shocks, the energy consumption of road freight transport is knocked out of equilibrium and needs 17 years and 4 months to adjust to the dynamically changing balance.

Research in this paper showed that is a strong correlation between road freight transport and its energy consumption with production in the construction, industry, and processing sectors. There are multidirectional dependencies between them, not only one-way but also in terms of feedback; some of the dependencies are also neutral, which is consistent with the findings on the hypothesis of connections between transport and the economy [52]. They are long-term and immediate. As in the work of other researchers, a significant impact of the shocks was transferred from one to another [72,97]. They overlap with feedback shocks that transform the direction of impact, especially in the industry and processing sectors. The results extend the literature [68,69,70,71] with contributions to the relationship between the energy consumption of road transport and other sectors of the drifting economy subject to shocks. So far, researchers have used the vector error correction model to study long-term relationships and the adjustment mechanism, but mainly in the area of the relationship between economic growth and energy consumption of the entire transport sector on a macroeconomic scale [55,56,58,59,62], while this paper deepened the research contribution at the mesoeconomic level concerning complementary sectors for road transport, i.e., the construction, industry, and processing sectors. Cognitively important is the mechanism of adapting to the equilibrium level in terms of factors shaping the dynamic equilibrium, after breaking out of it by various factors, including shocks. This is an important conclusion for the entire socio-economic system and management of the economy or the environment, as it gives a comparative scale of destabilization processes and the time to return to the stabilization path.

## 6. Conclusions

The rationalization of the energy intensity of road freight transport in Poland is undoubtedly an important issue that requires extended analysis. On the one hand, it is conditioned by economic, environmental, and social factors, and on the other hand, it affects the condition of the economy and the environment. Returning to the level of long-term equilibrium after breaking from it does not result from the nature of a growth-oriented economy, and therefore it is not a simple matter. It turns out that the equilibrium equations are not synchronized in time. There are complex, multidirectional, and complex cause–effect relationships between the energy intensity of road freight transport and other economic categories, and immediate causality overlaps with delayed causality. In addition, numerous structural shocks distort the forecasts for the energy consumption of road freight transport.

The presented analyses made it possible to identify the directions of rationalization of energy consumption in road freight transport in the light of environmental management, taking into account economic drift and leakage shocks in production in the construction, industry, and processing sectors. Based on the literature review, research gaps, methods, and variables were identified that were studied at the macroeconomic and mesoeconomic levels. As a result of the methodological review, it was possible to identify the research procedure and compare it with the conducted research. It was possible to diagnose a cognitive gap in the relationship between the energy consumption of road freight transport and production in the sectors serviced by this transport. Based on the results from the vector error correction model (VECM) and structural form (SVECM) obtained, rationalization paths for the energy consumption of road freight transport were identified, taking into account economic drift and long-term relations. The mechanism of adjustment to the equilibrium level was recognized by using equation forms, or leakage feedback shocks using IRF.

The conclusions of the research can be included in four points:

1. The criteria for rationalizing the energy intensity of road freight transport should correspond to the mechanism of macroeconomic adjustments, that is, result from both the co-integrating relations and the path of returning to the equilibrium level after precipitating the disturbance of the equilibrium state.
2. The mechanism of macroeconomic adjustments is a tool thanks to which it is possible to present the equation or equilibrium equations of the energy intensity of road freight transport, and therefore it has a significant meaning.
3. The energy intensity of road freight transport has no *a priori* character in the system of adjusting to the equilibrium; cointegrating relations play an important role here.
4. The shocks inherently throw a given system out of equilibrium, but they may fade out after some time, which means that the analyzed system of variables stabilizes. Nevertheless, the studied systems focused on structural shocks, which are sometimes desirable, because the system must be thrown out of balance to develop. Constant equilibrium is not desirable—it leads to stagnation.

An important factor in improving the energy consumption of road freight transport is the transfer of cargo handling from road transport to other modes (implementation of the assumptions of the shift paradigm), which will minimize the energy consumption of this type of transport. Transferring cargo handling to different modes of transport is an essential activity in the sphere of transport management and influences the economic and environmental order. On the other hand, an essential point of reference is the synchronization of the impact of shocks and an attempt to synchronously extinguish them through the implementation of transport policy tools (parametric and non-parametric). The analysis shows the coupling of production in the analyzed sectors of the economy with the energy consumption of road freight transport.

The studies carried out have limitations. They do not include other sectors that are also important for the development of the economy as a whole and could extend the modeling approach. In the future, the approach will be extended to take into account the COVID-19 pandemic crisis and inflationary shocks, which also have implications for fuel prices.

The research carried out is a novelty. In the search for rationalization directions for energy consumption, attention was paid to 21 overlapping relationships from Granger causality tests, the drift of the economy, and feedback shocks through the prism of production processes in the construction, industry, and processing sectors, the activity of which would not be possible without transport services. The research is interdisciplinary and mainly relates to the issues of economic management, environmental management, and transport management.

## Figures and Tables

**Table 2:** Summary statistics, using the observations January 2008–June 2016.

Variable	Mean	Median	Standard Deviation	Coefficient of Variance
EN	1.78 × 10^−4^	1.81 × 10^−4^	2.91 × 10^−5^	0.16
Qp	107.65	108.21	14.09	0.13
Qi	106.38	106.76	11.79	0.11
Qc	100.16	103.60	30.50	0.31
l_EN	−8.65	−8.62	0.17	0.02
l_Qp	4.67	4.68	0.13	0.03
l_Qi	4.66	4.67	0.11	0.02
l_Qc	4.56	4.64	0.33	0.07

Source: own calculations based on data from the OECD database [73].

**Table 3:** ADF test results for l_EN, l_Qp, l_Qi, l_Qc.

Test	l_EN [2]	l_Qp [10]	l_Qi [10]	l_Qc [2]
ADF Test Statistics	−3.0289	−2.8179	−3.0931	−1.7661

Note: The study took into account the optimal number of lag, drift, trend, and seasonal dummies. In square brackets, the optimal lag number determined by at least two information criteria is given, including Final Prediction Error (FPE), Akaike (AIC), Schwarz (SC), and Hannan–Quinn (HQ). The main information criterion based on which the optimal order was determined was FPE—it showed similar results in all cases with the use of AIC and in one case with the use of HQ. Source: own calculations based on data from the OECD database [73].

**Table 4:** Johansen test results for l_EN, l_Qp, l_Qi, l_Qc.

r0	LR	*p*-Value	90%	95%	99%
0	74.62	0.0039	60.00	63.66	70.91
1	35.20	0.2401	39.73	42.77	48.87
2	14.97	0.5841	23.32	25.73	30.67
3	4.90	0.6173	10.68	12.45	16.22

Note: r0 means rank number, LR means the Likelihood Ratio Test for the Cointetegration test, and *p*-value means a statistical measurement used to validate a hypothesis against observed data. The study took into account the optimal number of lag, drift, trend, and seasonal dummies. The optimal lag number is 3 and was determined using at least two information criteria: Final Prediction Error (FPE), Akaike (AIC), Schwarz (SC), and Hanna-Quinn (HQ). The main information criterion based on which the optimal order was determined was FPE—it showed similar results to HQ. Source: own calculations based on data from the OECD database [73].

**Table 5:** Specification of matrix B and the long-term interaction matrix.

Matrix B				Long-Term Interaction Matrix			
l_EN	l_Qc	l_Qi	l_Qp	l_EN	l_Qc	l_Qi	l_Qp
*	0	0	0	*	*	*	*
*	*	0	0	*	*	*	*
*	*	*	0	*	*	*	*
*	*	*	*	*	*	*	*

Note: zero (0) denotes disabled matrix elements; an asterisk (*) indicates enabled matrix elements, and desired values. Source: own elaboration.

**Table 6:** Diagnosis of cause-and-effect links between production in construction, industry, processing sectors, and energy intensity of road transport.

Causality in the Granger Sense		Immediate Causality in the Granger Sense	
Causes → Effects	Test Results	Causes → Effects	Test Results
l_Qc, l_Qi, l_Qp → l_EN	Test statistics l = 1.0503 *p*-value-F(l; 9, 276) = 0.4002	l_Qc, l_Qi, l_Qp → l_EN	Test statistics: c = 9.7431 *p*-value-Chi(c; 3) = 0.0209
Conclusions: Causality at a significance level below 10%, 5%, and 1% was not confirmed.		**Conclusions: There is causality at the 5% significance level.**	
l_EN → l_Qc, l_Qi, l_Qp	Test statistics l = 1.0781 *p*-value-F(l; 9, 276) = 0.3790	l_EN → l_Qc, l_Qi, l_Qp	Test statistics: c = 9.7431 *p*-value-Chi(c; 3) = 0.0209
Conclusions: Causality at a significance level below 10%, 5%, and 1% was not confirmed.		**Conclusions: There is causality at the 5% significance level.**	
l_EN, l_Qc → l_Qi, l_Qp	Test statistics l = 1.3677 *p*-value-F(l; 12, 276) = 0.1810	l_EN, l_Qc → l_Qp, l_Qp	Test statistics: c = 35.2643 *p*-value-Chi(c; 4) = 0.0000
Conclusions: Causality at a significance level below 10%, 5%, and 1% was not confirmed.		**Conclusions: There is causality at the 1% significance level.**	
l_Qi, l_Qp → l_EN, l_Qc	Test statistics l = 1.3751 *p*-value-F(l; 12, 276) = 0.1772	l_Qi, l_Qp → l_EN, l_Qc	Test statistics: c = 35.2643 *p*-value-Chi(c; 4) = 0.0000
Conclusions: Causality at a significance level below 10%, 5%, and 1% was not confirmed.		**Conclusions: There is causality at the 1% significance level.**	
l_EN, l_Qi → l_Qc, l_Qp	Test statistics l = 1.7780 *p*-value-F(l; 12, 276) = 0.0516	l_EN, l_Qi → l_Qc, l_Qp	Test statistics: c = 67.1629 *p*-value-Chi(c; 4) = 0.0000
**Conclusions: There is causality at the 10% significance level.**		**Conclusions: There is causality at the 1% significance level.**	
l_Qc, l_Qp → l_EN, l_Qi	Test statistics l = 1.5766 *p*-value-F(l; 12, 276) = 0.0979	l_Qc, l_Qp → l_EN, l_Qi	Test statistics: c = 67.1629 *p*-value-Chi(c; 4) = 0.0000
**Conclusions: There is causality at the 10% significance level.**		**Conclusions: There is causality at the 1% significance level.**	
l_EN, l_Qp → l_Qc, l_Qi	Test statistics l = 1.3390 *p*-value-F(l; 12, 276) = 0.1960	l_EN, l_Qp → l_Qc, l_Qi	Test statistics: c = 56.9971 *p*-value-Chi(c; 4) = 0.0000
Conclusions: Causality at a significance level below 10%, 5%, and 1% was not confirmed.		**Conclusions: There is causality at the 1% significance level.**	
l_Qc, l_Qi → l_EN, l_Qp	Test statistics l = 2.1256 *p*-value-F(l; 12, 276) = 0.0156	l_Qc, l_Qi → l_EN, l_Qp	Test statistics: c = 56.9971 *p*-value-Chi(c; 4) = 0.0000
**Conclusions: There is causality at the 5% significance level.**		**Conclusions: There is causality at the 1% significance level.**	
l_EN, l_Qi, l_Qp → l_Qc	Test statistics l = 1.6239 *p*-value-F(l; 9, 276) = 0.1081	l_EN, l_Qi, l_Qp → l_Qc	Test statistics: c = 29.2616 *p*-value-Chi(c; 3) = 0.0000
Conclusions: Causality at a significance level below 10%, 5%, and 1% was not confirmed.		**Conclusions: There is causality at the 1% significance level.**	
l_Qc → l_EN, l_Qi, l_Qp	Test statistics l = 1.7846 *p*-value-F(l; 9, 276) = 0.0710	l_Qc → l_EN, l_Qi, l_Qp	Test statistics: c = 29.2616 *p*-value-Chi(c; 3) = 0.0000
**Conclusions: There is causality at the 10% significance level.**		**Conclusions: There is causality at the 1% significance level.**	
l_EN, l_Qc, l_Qp → l_Qi	Test statistics l = 1.6728 *p*-value-F(l; 9, 276) = 0.0953	l_EN, l_Qc, l_Qp → l_Qi	Test statistics: c = 56.8563 *p*-value-Chi(c; 3) = 0.0000
**Conclusions: There is causality at the 10% significance level.**		**Conclusions: There is causality at the 1% significance level.**	
l_Qi → l_EN, l_Qc, l_Qp	Test statistics l = 2.2849 *p*-value-F(l; 9, 276) = 0.0174	l_Qi → l_EN, l_Qc, l_Qp	Test statistics: c = 56.8563 *p*-value-Chi(c; 3) = 0.0000
**Conclusions: There is causality at the 5% significance level.**		**Conclusions: There is causality at the 1% significance level.**	
l_EN, l_Qc, l_Qi → l_Qp	Test statistics l = 2.3986 *p*-value-F(l; 9, 276) = 0.0124	l_EN, l_Qc, l_Qi → l_Qp	Test statistics: c = 54.2728 *p*-value-Chi(c; 3) = 0.0000
**Conclusions: There is causality at the 5% significance level.**		**Conclusions: There is causality at the 1% significance level.**	
l_Qp → l_EN, l_Qc, l_Qi	Test statistics l = 1.6532 *p*-value-F(l; 9, 276) = 0.1003	l_Qp → l_EN, l_Qc, l_Qi	Test statistics: c = 54.2728 *p*-value-Chi(c; 3) = 0.0000
Conclusions: Causality at a significance level below 10%, 5%, and 1% was not confirmed.		**Conclusions: There is causality at the 1% significance level.**	

Source: own calculations based on data from the OECD database [73].

**Table 7:** Estimation results for matrix B and long-term interaction matrix.

Matrix B				Long-Term Interaction Matrix			
0.0700	0.0000	0.0000	0.0000	0.0522	−0.0021	0.0030	0.0019
0.0040	0.0737	0.0000	0.0000	−0.0092	0.1488	−0.0540	0.1453
−0.0100	0.0301	0.0369	0.0000	−0.0055	0.0227	0.0148	0.0086
−0.0111	0.0354	0.0376	0.0054	−0.0075	0.0374	0.0070	0.0243

Source: own calculations based on data from the OECD database [73].

## Data Availability

Data are contained within the article. To estimate the analyzed results, the authors used raw data from the databases included in the references listed as [73].
